# Potential of essential fatty acid deficiency with extremely low fat diet in lipoprotein lipase deficiency during pregnancy: A case report

**DOI:** 10.1186/1471-2393-4-27

**Published:** 2004-12-20

**Authors:** Elaine C Tsai, Judy A Brown, Megan Y Veldee, Gregory J Anderson, Alan Chait, John D Brunzell

**Affiliations:** 1 Department of Medicine, University of Washington, Seattle, Washington, USA; 2 Veterans Affairs Puget Sound Health Care System, 1660 S. Columbian Way (152E), Seattle, Washington, USA; 3 Oregon Health Sciences University, Portland, Oregon, USA

## Abstract

**Background:**

Pregnancy in patients with lipoprotein lipase deficiency is associated with high risk of maternal pancreatitis and fetal death. A very low fat diet (< 10% of calories) is the primary treatment modality for the prevention of acute pancreatitis, a rare but potentially serious complication of severe hypertriglyceridemia. Since pregnancy can exacerbate hypertriglyceridemia in the genetic absence of lipoprotein lipase, a further reduction of dietary fat intake to < 1–2% of total caloric intake may be required during the pregnancy, along with the administration of a fibrate. It is uncertain if essential fatty acid deficiency will develop in the mother and fetus with this extremely low fat diet, or whether fibrates will cross the placenta and concentrate in the fetus.

**Case presentation:**

A 23 year-old gravida 1 woman with primary lipoprotein lipase deficiency was seen at 7 weeks of gestation in the Lipid Clinic for management of severe hypertriglyceridemia that had worsened with pregnancy. While on her habitual fat intake of 10% of total calories, her pregnancy resulted in an exacerbation of the hypertriglyceridemia, which prompted further restriction of fat intake to < 2% of total calories, as well as administration of gemfibrozil at a lower than average dose. The level of gemfibrozil, as the active metabolite, in the venous and arterial fetal cord blood was within the expected therapeutic range for adults. The clinical signs and a biomarker of essential fatty acid deficiency, namely the ratio of 20:3 [n-9] to 20:4 [n-6] fatty acids, were closely monitored throughout her pregnancy. Despite her extremely low fat diet, the levels of essential fatty acids measured in the mother and in the fetal blood immediately postpartum were normal. Normal essential fatty acid levels may have been achieved by the topical application of sunflower oil.

**Conclusions:**

An extremely low fat diet in combination with topical sunflower oil and gemfibrozil administration was safely implemented in pregnancy associated with the severe hypertriglyceridemia of lipoprotein lipase deficiency.

## Background

Primary lipoprotein lipase (LPL) deficiency is a rare autosomal recessive disorder characterized by severe hypertriglyceridemia, due to the accumulation in plasma of chylomicrons and very low density lipoproteins (VLDL) that result from the absence of LPL activity [1]. The estimated frequency of this disorder is <1 per million with the carrier frequency at about 1 in 500. Clinically significant hypertriglyceridemia usually manifests early in childhood with dietary fat intolerance, including recurrent episodes of abdominal pain and acute pancreatitis, failure to thrive, eruptive xanthoma and hepatosplenomegaly. Very severe hypertriglyceridemia during pregnancy can occur and is associated with significant maternal morbidity and fetal mortality [2-6]. Overproduction of hepatic VLDL in the presence of decreased LPL activity contributes to the marked increase in plasma triglyceride (TG) levels during pregnancy [7,8].

The management of severe hypertriglyceridemia in a pregnant patient with LPL deficiency is directed toward preventing pancreatitis in the mother and delivery of a healthy infant. Lowering of plasma TG in the prevention of pancreatitis is managed primarily by dietary fat restriction, but additional TG lowering may be required and has been reported with use of fibrates, such as gemfibrozil [6,9,10]. Two major questions arose during the treatment aimed at lowering the severe hypertriglyceridemia in this pregnant LPL-deficient patient. First, would essential fatty acid (EFA) deficiency develop in the mother and fetus as a result of severe maternal dietary fat restriction? Second, would gemfibrozil cross the placenta and concentrate in the fetus? The strategies utilized to prevent EFA deficiency and the fetal nutritional information obtained from studies at birth will address these questions and concerns.

## Case presentation

### Clinical history

The proband presented to her pediatrician at age 3 month with failure to thrive. She was evaluated at the Children's Hospital Medical Center in Seattle, where an elevated TG level of 14,000 mg/dl (158 mmol/L) suggested hyperchylomicronemia with LPL deficiency. Plasma post-heparin LPL activity was absent, consistent with defective catabolism of TG rich particles. Further study at the University of Washington of her post-heparin plasma at age 19 revealed absent LPL activity due to a defective LPL protein, while her hepatic lipase activity was normal [11]. She was a compound heterozygote with two missense mutations in the LPL gene (Trp86-Arg/His136-Arg) [12]. On a self-selected low fat diet (< 20% of dietary calories) she was able to maintain her TG < 1000 mg/dl (11.3 mmol/L) throughout a healthy and normal childhood and adulthood. Because of her excellent compliance with low dietary fat intake and active physical lifestyle, she had never developed clinical pancreatitis. Throughout the years, there were few episodes of mid epigastric abdominal discomfort that subsided with short periods of fasting. She had developed eruptive xanthoma briefly when oral contraceptives were used.

### Pregnancy course

**Figure 1:**
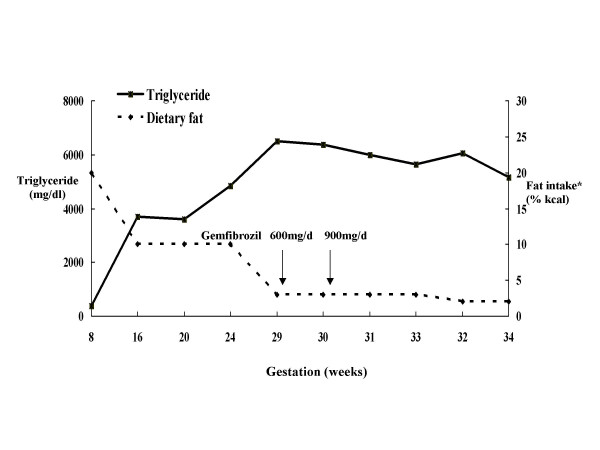
Serum triglyceride level and corresponding dietary fat intake and gemfibrozil administration during pregnancy *Dietary fat was expressed as % of total caloric intake.

At the age of 23, the proband presented at week 7 of gestation for management of anticipated worsening of hypertriglyceridemia in pregnancy. She had been in excellent physical condition and had continued her routine 10–20% fat diet during the first trimester (Figure 1). Her TG was 396 mg/dl (4.5 mmol/L) at week 7 of gestation. When she retuned to the University of Washington Medical Center (UWMC) 5 weeks later, her TG levels had started to rise and despite further restriction of dietary fat to < 10% of calories, the level had risen to 3705 mg/dl (41.9 mmol/L) by week 16. At week 28, she developed her first episode of mid epigastric abdominal pain without elevated serum amylase or pancreatic lipase levels, consistent with subclinical pancreatitis. Remission of the symptoms occurred within 2–3 days of a near zero dietary fat intake as an outpatient. Subsequent reduction to less than 2% of dietary fat was implemented with a liquid formula by the following week, to decrease the risk of recurrent abdominal pain in the setting of extremely elevated TG levels (3000–6000 mg/dl [33.9–67.8 mmol/L]). Gemfibrozil at a low dose of 300 milligram (mg) twice a day was also initiated at week 29 to prevent a further upward trend in TG in the third trimester. The dosage was increased to 300 mg three times a day a week later. This appeared to stabilize her TG in the 5000–6000 mg/dl (56.5 – 67.8 mmol/L) range until week 34 when she developed severe abdominal pain. Initial evaluation at her local hospital revealed a TG level of 6,050 mg/dl (68.4 mmol/L) and elevated pancreatic lipase (680 IU/dl) and amylase (1336 IU/L). She was transferred and admitted to the UWMC and placed on intravenous fluids. Two days later, her pancreatitis subsided and she was placed back on the <2% fat diet and 900 mg/day of gemfibrozil. A second episode of pancreatitis a few days later prompted re-admission to UWMC for labor induction at the 35^th ^week of gestation. A 5 lb 3 oz baby girl with a 5-minute Apgar score of 9 was delivered vaginally. A short time after the delivery, the baby was briefly intubated for about 48 hours due to respiratory distress but did well subsequently. The patient's plasma TG rapidly decreased to 2015 mg/dl (22.8 mmol/L) within the first postpartum 24 hours, accompanied by improved abdominal symptoms. Resumption of low fat solid food brought back the symptoms of pancreatitis and she was placed back on the IV fluids followed by a more gradual incremental introduction of oral intake. Along with 1,200 mg/day of gemfibrozil, she had complete resolution of abdominal symptoms by postpartum day-8 and eventual resolution two weeks after discharge of peri-pancreatic fluid accumulation demonstrated by CT imaging studies. Now, 11 years later, the proband and her daughter are both healthy and doing well. The proband's TG levels are back to baseline and stable on 10–20% fat diet. Her daughter has had normal TG and cholesterol levels on regular diet.

Eruptive xanthomas, which are associated with hypertriglyceridemia, developed on the proband's buttocks at week 20 and subsequently spread to the upper arms and medial aspects of thighs as her TG levels rose. Peculiarly, palmar xanthoma, typical of remnant removal disease (type III hyperlipidemia), also developed at week 27. A concomitant *ex vivo *investigation of the mechanisms contributing to palmar xanthoma in the proband, who has an apo E 4/E3 phenotype, revealed an enhanced macrophages uptake of the TG rich lipoproteins as a result of an unusual enrichment of these lipoproteins with apo E during pregnancy [13].

### Gestational EFA profiles

**Figure 2:**
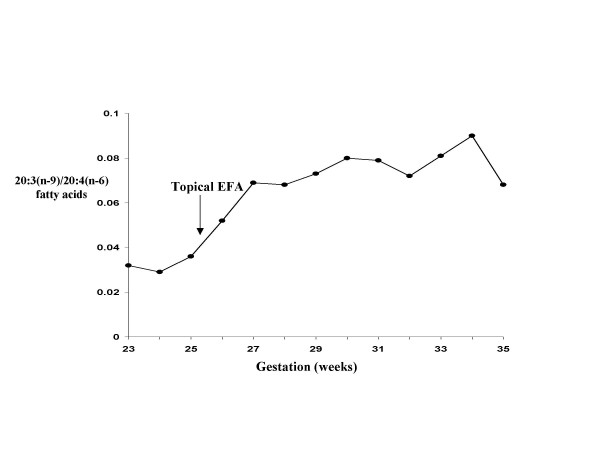
Essential fatty acid profile in maternal blood EFA: essential fatty acids, from sunflower seed oil. Measurement was made in the phospholipid fractions. 20:3(n-9): eicosatrienoic acid (ETA). 20:4(n-6): arachidonic acid (AA).

Because of concern for unfavorable fetal neurological development due to EFA deficiency, EFA profile was monitored in the mother at each visit starting at gestational week 23. The initial analyses were performed at the Clinical Nutrition Research Unit (CNRU), Harborview Medical Center campus of the University of Washington. After separation from cells, the fatty acids from the phospholipid fraction of the plasma were extracted and subsequently measured by capillary gas chromatography. In addition to the total amount and % of each FA, the ratio of eicosatrienoic acid (ETA, 20:3(n-9)) to arachidonic acid (AA, 20:4(n-6)) was calculated (Figure 2). This ratio was used as an index to the patient's EFA status. By week 26, the ratio had risen from 0.032 to 0.052 (Fig 2), suggesting a trend to a less EFA abundant state [14]. Topical application of sunflower oil containing large amounts of polyunsaturated fatty acids (PUFA) was initiated as a non-oral route for supplementing EFA because of its reported success in EFA deficient subjects [15]. With 460 mg per day of sunflower seed oil (approximately 240 mg of linoleic acid) applied to her arms and trunk, her EFA profile appeared to improve with the ratio stabilizing at 0.08 at 31 weeks. A peak to a ratio of 0.09 occurred at week 34 possibly due to irregular uses of topical PUFA (Figure 2).

**Figure 3:**
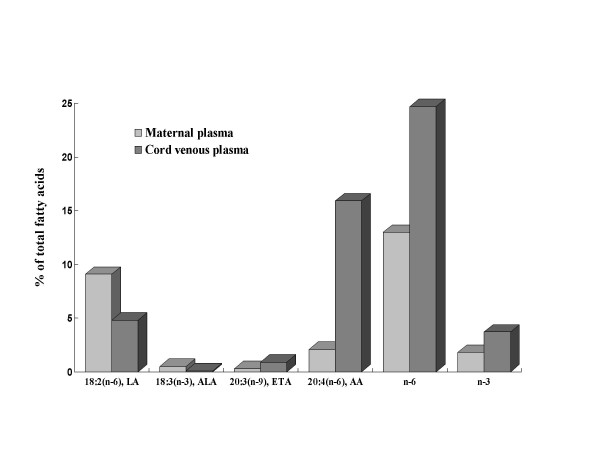
Fatty acid composition in maternal and cord plasma LA: linoleic acid. ALA: α-linolenic acid. ETA: eicosatrienoic acid. AA: arachidonic acid.

Immediately postpartum, placental fetal blood and maternal plasma was obtained for total fatty acid analysis (Figure 3). These FA were measured by capillary gas/liquid chromatography at the Oregon Health Sciences University and expressed as % of total FA. In spite of low levels of n-6 and n-3 fatty acids in maternal blood and similarly decreased levels of PUFA precursors (linoleic [LA] and α-linolenic acid [ALA]) in cord blood samples compared to the reported normal reference range [16], there were abundant long chain PUFA (such as arachidonic acid [AA]) in the fetal circulation. This suggested that either the topical application of sunflower seed oil during the late stage of pregnancy prevented EFA deficiency or that the fetus had increased capacity for obtaining EFA from the mother.

### Gemfibrozil in fetal circulation

**Figure 4:**
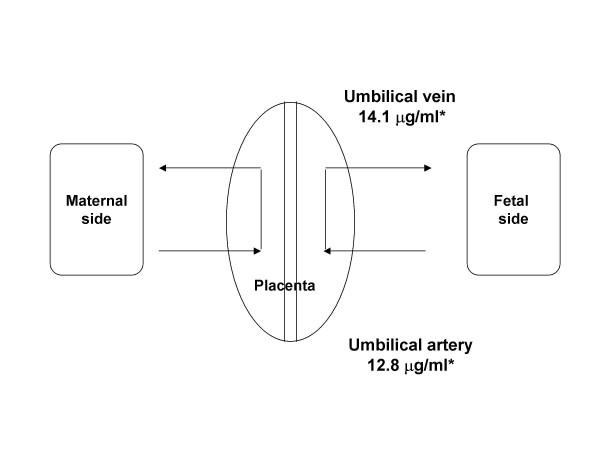
Gemfibrozil metabolite III levels in the fetal cord blood *Ref. range: 0.5 – 40 μg/ml

To examine whether there might be excessive accumulation of gemfibrozil in the newborn baby, fetal cord blood was obtained at the time of delivery and gemfibrozil levels and its active compound, metabolite III, were measured. The analysis was performed as a courtesy of the Research Lab at the Parke-Davis Pharmaceutical (Ann Arbor, Michigan) by high performance liquid chromatography (HPLC) and revealed similar concentrations of the drug and its active metabolites in both umbilical vein and artery, which were within the normal reference range for adults (Figure 4).

## Conclusions

Children with primary LPL deficiency can be effectively managed on fat-restricted diets and grow normally into adulthood. However, they can present with extreme elevation of TG levels with serious acute pancreatitis. This LPL-deficient subject developed severe hypertriglyceridemia in early pregnancy, with eruptive xanthomas and pancreatitis. With the diligent efforts from the patient, her family, and a team of specialists in lipid metabolism, dietetics, high-risk obstetrics and gastroenterology, a successful outcome was achieved. Outcome goals were clearly set at the onset of her pregnancy care, including nutritional management of the expected rise in triglyceride levels associated with the estrogen surge of pregnancy to prevent acute pancreatitis, and avoidance of clinical EFA deficiency in both the mother and the fetus.

### Pregnancy and hypertriglyceridemia

Pregnancy-induced hypertriglyceridemia is estimated to be the cause in 4–6% of all pancreatitis cases during pregnancy, while most cases result from cholelithiasis [4]. Hypertriglyceridemia-related pancreatitis in pregnancy also has been reported due to other causes of severe hypertriglyceridemia [17]. Successful management requires early detection of signs and symptoms of acute pancreatitis often accompanied by increases in serum lipase and amylase levels and characteristic findings in imaging studies. Once the pancreatitis is suspected, these individuals should be admitted for aggressive medical management including intravenous hydration concurrent with no oral intake of solids or liquids. Obstructive processes in the biliary system need to be ruled out specifically since treatment modalities are quite different.

### Pregnancy and LPL deficiency

Pregnancy is a well known situation in which the physiologic estrogen surge profoundly alters the TG-rich lipoprotein metabolism, resulting in a gradual rise in TG levels over the course of non-complicated pregnancy, peaking at the level of 200–300 mg/dl (2.26 – 3.39 mmol/L) at term [17]. During the first two trimesters of pregnancy, adipose fat storage, as maternal fuels, occurs in preparation for an active transfer of maternal glucose, amino acids, and free fatty acids (FFA) across the placenta for accelerate fetal growth in late phase of gestation [18]. In late gestation, adipose tissue lipolysis is greatly augmented generating FFA and glycerol, for further hepatic VLDL production, contributing to the flux of circulating TG-fatty acids in pregnancy [18,19]. Greater concentration of chylomicrons from dietary fat as a result of maternal hyperphagia in late pregnancy also contributes to the circulating TG-rich lipoprotein pool [18,19], and provides alimentary substrates for VLDL production [20,21]. LPL activities in the liver, heart, and particularly adipose tissue are, however, reduced by an estimated total of 85% [19,22] in late gestation. Concomitantly, clearance of circulating TG-rich lipoproteins is reduced in late pregnancy. Hepatic lipase activity is decreased as well and could explain the observation of parallel TG-enrichment of LDL and high-density lipoproteins (HDL) particles during normal gestation. All these changes take place to ensure a stable supply of fuel substrates across the placenta for normal fetal development while preserving maternal metabolic homeostasis [18,19].

### Very low fat diet and EFA deficiency

Arachidonic acid [AA, 20:4(n-6)], an important precursor of the prostaglandin compounds, cannot be synthesized de novo from FFA in mammals and must be derived from another EFA in the diet, namely linoleic acid [LA, 18:2(n-6)]. In the case of life long low oral fat intake, as in our patient, clinical EFA deficiency might occur with depletion of n-3 and n-6 FA stored in adipose tissue. Therefore, her source of EFA would be entirely from recent dietary intake and deficiency might occur sooner than in individuals with normal LPL and abundant EFA storage [23]. Eicosatrienoic acid [ETA, 20:3(n-9)], on the other hand, is not an EFA because it can be synthesized in mammals from palmitic acid [16:1(n-9)]. In the event of diminishing pool of both n-3 and n-6 fatty acids due to absence or deficiency in the diet, more ETA are produced and the amount parallels the degree of deficiency [24-26]. EFA deficiency syndrome commonly results from a combined deficiency in both n-3 and n-6 fatty acids. A ratio of ETA to AA > 0.2, is suggestive of EFA deficiency [24-26]. Clinical manifestations in EFA deficiency are unusual on a diet containing > 2% of the calories as linoleic acid [27]. While the clinical symptoms of dryness and desquamation of the skin are annoying at best, a more serious consequence could be impaired fetal brain and visual development. The proband did not develop signs of clinical EFA deficiency, nor did the ratio of 20:3(n-9) to 20:4(n-6) exceed 0.2 at any stage of her pregnancy, although an upward trend did occur. Additionally, the report that infants fed a formula low in EFA grew poorly and developed multiple medical complications was a concern [28]. Several reports have documented a reversal of biochemical and clinical manifestations of EFA deficiency in infants and adults by cutaneous administration of EFA-rich oil, such as sunflower oil [29-35]. Therefore, application of sunflower oil to the proband's skin was initiated at week 25 and may have had prevented progression of EFA deficiency in mother, as suggested by the stabilization of the 20:3(n-9) to 20:4(n-6) ratio. Surprisingly, we found low levels of n-3, n-6, and PUFA precursor levels in the cord blood taken during the delivery, and yet there was abundant long chain PUFA in the infant circulation. This would suggest that other adaptive mechanisms were involved in maintaining the critical levels of long chain EFA in fetal circulation in the face of inadequate maternal supply.

### Use of gemfibrozil in LPL deficiency

Use of TG lowering drugs, such as gemfibrozil (a fibrate), can be used to directly lower the triglyceride level in the prevention of acute pancreatitis. Pregnancy induces hepatic production of TG-rich VLDL and may respond to fibrates through inhibition of hepatic production of VLDL. Gemfibrozil, which is an FDA category C drug, has not been observed to be associated with adverse drug effects in reports of pregnancy-related severe hypertriglyceridemia [6,10,36,37]. During the last few weeks of her gestation low dose gemfibrozil in our subject seemed to have stabilized her TG level (Fig 1), which might otherwise have continued to rise due to the estrogen effect on hepatic VLDL production in the third trimester. A lower than usual dose of gemfibrozil was used due to the concern for excess placental transfer of its metabolites that has been reported in pregnant cats [38]. Analysis of the parent compound and metabolites did not detect excessive accumulation in the fetal cord circulation in contrast to the reports in animal models. While this observation needs to be independently confirmed, adverse drug effects in the infants born to mothers on gemfibrozil or other fibrates have not been reported. Moreover, gemfibrozil has been used and appears to be free of short-term side effects in pediatric populations [39-42]. Therefore, low dose gemfibrozil may be safe for use during the last trimester in hyperlipidemic patients at high risks for acute pancreatitis.

In conclusion, a successful pregnancy outcome was achieved in our LPL deficient patient, confirming previous reports [6,43] that aggressive lipid lowering strategies under the supervision of experienced health care providers works in this high risk setting. Although the patient developed pancreatitis during her pregnancy, the use of an extremely low fat diet together with a fibrate helped limit the increase in the triglycerides, and her pancreatitis was neither life threatening nor adversely affected fetal survival. Sunflower oil applied topically may have helped prevent EFA deficiency in both the mother and fetus. Use of gemfibrozil did not appear to have any adverse effect on the child. Thus, the use of these two therapeutic approaches appears safe and appropriate in the medical management of pregnancy-associated severe hypertriglyceridemia, where EFA deficiency and recurrent pancreatitis are major concerns.

## Competing interests

The author(s) declare that they have no competing interests.

## Authors' contributions

ECT was a senior fellow in Metabolism, Endocrinology and Nutrition and drafted the manuscript. JAB was the dietitian. Both MSV and GJA contributed to the measurement of fatty acids. GJA was also a consulting scientist in fatty acid metabolism. AC and JDB were the faculty associated with the case at the UW GCRC. JDB conceived of the research, supervised the fellow, and coordinated the manuscript revisions.

## Pre-publication history

The pre-publication history for this paper can be accessed here:


